# Correction to: ‘Giant Jurassic dragon lacewing larvae with lacustrine palaeoecology represent the oldest fossil record of larval neuropterans’ (2023) by Du *et al.*

**DOI:** 10.1098/rspb.2023.0411

**Published:** 2023-03-29

**Authors:** Xuheng Du, Kecheng Niu, Tong Bao

*Proc. R. Soc. B* **290**, 20222500. (Published online 15 February 2023). (https://doi.org/10.1098/rspb.2022.2500)

After publication of the article [1], it was brought to our attention that the genus name we proposed for the new fossil is a junior homonym of *Palaeoneurorthus* Wichard, 2009 [2]. In accordance with ICZN article 60.3, we herein propose substitute names for the genus and the species, with the same type designations and diagnoses as the original publication. We thank the colleagues who pointed out this issue.

***Girafficervix***, nomen novum

in substitution for *Palaeoneurorthus* Du, Niu & Bao

*Type species: Girafficervix baii* Du, Niu & Bao*,* typification by monotypy.

*Diagnosis:* as species.

*Gender:* feminine.

*Etymology*: the name is compounded from the genus name *Giraffa*, meaning giraffe, and *cervix*, Latin meaning neck, in reference to the specimen's long neck.

***Girafficervix baii*, nomen novum**, holotype YLNHM01098, paratype NIGP201165 in substitution for *Palaeoneurorthus baii* Du, Niu & Bao

Refer to Du, Niu & Bao (2023) [1] for details on the holotype, diagnoses and etymology.
